# A case of bilateral oophorectomy in a 40-day-old female due to a misdiagnosis of complete androgen insensitivity syndrome

**DOI:** 10.1097/MS9.0000000000003830

**Published:** 2025-09-30

**Authors:** Ahmad Chreitah, Bashar Abo Kaf, Omar Aljanati, Zeina Alkilany, Maya Issa, Ibraheem Jraikoos

**Affiliations:** a Faculty of Human Medicine, Tishreen University, Latakia, Syria; b Department of Pediatrics, Tishreen University, Latakia, Syria; c Department of Pediatrics, Tishreen University Hospital, Latakia, Syria

**Keywords:** artificial pubertal induction, bilateral inguinal masses, CAIS, oophorectomy, secondary sexual characteristics

## Abstract

**Introduction and importance:**

Bilateral oophorectomy in females is rarely performed in the pediatric population due to its serious physical and mental long-term consequences on both the patients and their parents, which make it very complicated to manage.

**Case presentation:**

A 13-year-old female was referred to our pediatric clinic for evaluation of short stature and the absence of secondary sexual characteristics. As part of her past medical history, she underwent a bilateral oophorectomy that was performed along with a bilateral hernia repair at the age of 40 days due to misdiagnosis as complete androgen insensitivity syndrome (CAIS). The patient was initially started on transdermal hormonal replacement therapy and then replaced by oral treatment. A follow-up was planned every 6 months with a good response to the treatment regarding the development of secondary sexual characteristics and the satisfactory final height.

**Clinical discussion:**

The girl underwent bilateral oophorectomy after a misdiagnosis of CAIS without proper diagnostic procedures, leading to medicolegal and ethical issues. Karyotyping is necessary for the diagnosis of CAIS. The adolescent received hormonal replacement therapy and had a follow-up of her growth and sexual development. The concept of oocyte donation was also discussed with the family.

**Conclusion:**

Performing a karyotype and a complete hormonal workup is essential in any suspicion of CAIS in a phenotypically female infant to avoid making such an error. Performing the gonadectomy after sexual maturation is achieved in CAIS patients.

## Introduction

Oophorectomy is the surgical removal of the ovary; it can be either unilateral or bilateral. A bilateral oophorectomy BO is very rarely performed, especially among pediatric patients due to its long-term physical and psychological complications^[1]^. Complete androgen insensitivity syndrome (CAIS) is a rare condition where 46,XY individuals show complete unresponsiveness to the effects of androgens leading to a female phenotype and two normal functioning undescended testes. A common manifestation of CAIS is bilateral inguinal hernias; the management usually includes the surgical removal of the gonads and the long-term hormonal replacement therapy (HRT)^[2]^.

**HIGHLIGHTS**

- Oophorectomy is the surgical removal of the ovary; it can be either unilateral or bilateral. A bilateral oophorectomy BO is very rarely performed, especially among pediatric patients.
- Before deciding to perform any surgical procedure to remove a bilateral oophorectomy, the diagnosis should be carefully verified.

Here, we present a case of bilateral oophorectomy in a 40-day-old infant, performed without a definitive diagnosis or any investigations. This procedure in a child of this age exposes the child to psychological and physical effects throughout her life and to the need for long-life replacement therapy and follow-up.

## Case presentation

**Figure 1.**
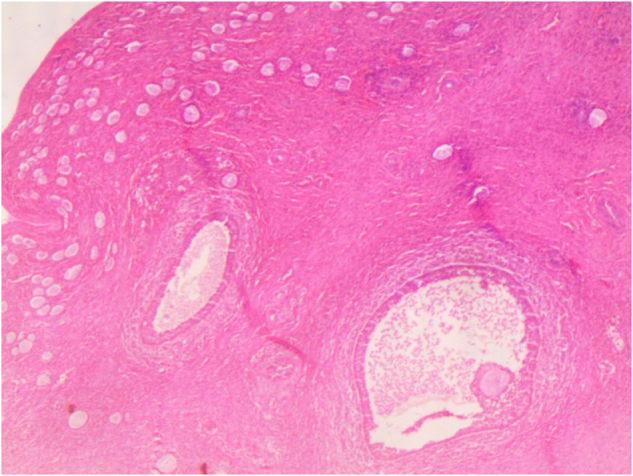
Normal ovaries.

**Figure 2.**
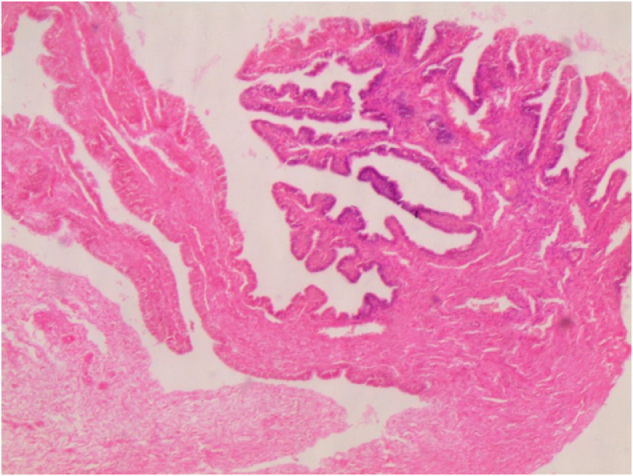
Normal fallopian tubes.

A 13-year-old female was referred to our pediatric endocrinology clinic for an evaluation concerning the absence of secondary sexual characteristics and short stature. She is the third child of a family of good socioeconomic status. She was the offspring of nonconsanguineous parents, born at 33 weeks of a twin pregnancy, and she is the first twin. Her birth weight was 1400 g, and she was hospitalized in the neonatal intensive care unit for 10 days. At the age of 40 days, she had a surgical consultation for bilateral inguinal masses. The surgeon made the diagnosis of CAIS and performed a bilateral surgical resection, with no karyotype, magnetic resonance imaging (MRI), or hormonal workup. Histopathology of the two resected masses showed two normal ovaries (Fig. 1) with fallopian tubes (Fig. 2).

At admission, she was conscious, anxious, and thin, with no facial dysmorphia. The blood pressure was 110/70 mmHg, the heart rate was 90 bpm, and the respiratory rate was 20 bpm. Based on Centers for Disease Control and Prevention (CDC) charts, her weight was 28 kg (3 SD below the mean), her height was 136 cm (3 SD below the mean), and her Body mass index (BMI) was 15.1 (4.1 percentile). The rest of her physical examination was unremarkable. The girl’s appetite is not good; she is a vegetarian, and her school and social performance are poor. She was considerably shorter than her twin sister, who was having a normal pubertal process at the time. While secondary sexual characteristics had not yet appeared in our patient (Tanner 1). In the family history, the mother had her first menses at 12 years, and her twin sister had her first menses at 13 years. Her father’s height is 172 cm, her mother’s height is 152 cm, and her expected height is 158.5 cm. The initial hormonal workup showed an elevation in follicle-stimulating hormone and luteinizing hormone (LH) hormones (Table 1).

**Table 1:** Biochemical workup

Test	Result	Normal range	Test	Result	Normal range
TSH	2.2 mIU/L	0.3–5 mIU/L	Anti-TTg	2.7 U/mL	<4.0 U/mL
CA	8.8 mg/dL	8.8–10.8 mg/dL	HP antigens	Negative	
P	5.6 mg/dL	4.5–6.5 mg/dL	Hg	11.9 g/dL	11–15.5 g/dL
ALP	421 IU/L	130–340 IU/L	Urine	Normal	
FSH	12.12 IU/L	0.6-6 IU/L	Cortisol 8 AM	18 µg/dL	5–25 µg/dL
LH	31.8 IU/L	0.03–3.9 IU/L	Igf1	N	
ACTH	23 pg/mL	9–52 pg/mL	Estradiol	26 pg/mL	

CA, calcium; P, phosphorus; ALP, alkaline phosphatase; FSH, follicle-stimulating hormone; LH, luteinizing hormone; ACTH, adrenocorticotropic hormone; HP, *Helicobacter pylori*; Hg, hemoglobin; IGF1, insulin-like growth factor 1.

The table shows a decrease in gonadotropins (FSH and LH) and estradiol in the lower limits and an increase in alkaline phosphatase.

Bone densitometry showed osteopenia. Artificial pubertal induction started at the age of 13.3 years using transdermal estrogen therapy, which began with a dose of 0.0125 mg/day, doubled it every 6 months to a maximum dose of 0.625 mg/day, which was later replaced with oral estrogen due to its high cost and unavailability. Clinical and hormonal evaluations were performed every 6 months, including sexual characteristics and liver function tests.

After 24 months of treatment with estrogen her secondary sexual characteristics were fully developed (Tanner 4), her final height was 155 cm (−1 SD) close to expected height, 48 kg (−0.5 SD), and BMI 20 (51 percentile). Progesterone therapy of 200 mg/day for 12 days per month was initiated to oppose the effect of estrogen. Abdominal and pelvic ultrasound showed a uterine cavity 28 mm long, 14 mm wide, and an absence of ovaries.

## Discussion

This is a case of bilateral inguinal hernia but underwent to bilateral oophorectomy due to being incorrectly diagnosed with CAIS without following the correct diagnostic procedures. To the best of our knowledge, this is the first case of this nature to have been documented in the medical literature. The importance of this anecdotic case comes from its unique nature and challenging long-term management after this decision taken by the surgeon, which caused medicolegal issues, ethical dilemmas, lifelong treatment with its side effects, psychosocial problems, and infertility.

Primary inguinal hernia occurs in 1–5% of all newborns and 9–11% of those born prematurely. Bilateral hernia is more common in premature and low-birth-weight infants^[3]^.

The incidence of inguinal hernia in CAIS is higher than in the general population^[2]^.

The androgen insensitivity syndrome is caused by mutations in the androgen receptor gene. The complete form (CAIS) is characterized by XY sex reversal and normal female external genitalia, with the testis commonly located in the abdomen or inguinal canal^[4]^, with an estimated prevalence ranging from 1:20 000 to 1:100 000 male subjects^[2]^.

Karyotyping is necessary to make the diagnosis of CAIS in apparently normal females with unilateral or bilateral inguinal hernia^[2]^. A definite diagnosis of CAIS can only be made with genetic testing since clinical features, and lab values can be inadequate^[5]^. Serum testosterone concentration is in the usual adult male range, normal to elevated LH, and inhibin B in the mid-normal male reference range^[6]^.

Gonadectomy in CAIS patients is usually undertaken to reduce the risk of testicular cancers, which are uncommon among these patients before sexual maturation. Additionally, a spontaneous pubertal process is usually expected to occur in these patients. That is why the procedure is usually delayed until after sexual maturation occurs even in the presence of a bilateral hernia^[7]^. Ovarian surgery is a rare procedure in girls less than 20 years of age, and since most of these lesions are benign, ovarian preserving operations should be performed whenever feasible. Ovarian torsion is one of the most common gynecologic surgical emergencies and may affect females of all ages^[8]^.

Surgical intervention is indicated for ovarian torsion and suspected cancer and in some cases of rupture. In particular, ovarian cyst-associated torsion, which is an emergency requiring early intervention, is more common in young girls compared with adults^[9]^.

In addition to the loss of fertility and the failure to develop normal secondary sexual characteristics the loss of ovarian functions at such a young age can easily disturb normal physiology. Unmanaged BOs affect different systems of the human body, leading to an increased incidence of osteoporosis, neurological diseases, and coronary artery disease. In addition, the abrupt loss of ovarian functions severely affects the mental well-being of the patient, which is reduced by HRT, while maintaining a normal level of hormones and developing secondary sexual characteristics^[1]^.

HRT should be individualized according to each patient’s requirements and traits, though the initiation should be around 11 years of age. Many trials have compared the use of transdermal and oral estrogens, with most evidence leaning toward transdermal 17 β-estradiol as it leads to better therapeutic outcomes and fewer side effects. Both routes of administration have comparable metabolic effects, yet transdermal estrogen is more suitable for pubertal induction, leading to closer to normal uterine size and bone accrual. Progesterone should be added later in the process to avoid the dangers of unopposed estrogen^[10]^.

Follow-up every 6 months is very important in cases of pubertal induction. For monitoring secondary sexual characteristics, growth, bone density, uterine size, and blood pressure, liver function, coagulative state, lipid panels, and blood glucose should also be monitored. A routine neurological evaluation should take place since the earlier onset of neurological diseases is expected^[10]^.

The family was referred for psychological counseling to follow up on the child and the mother, who showed a somewhat bad mood. Medical literature encourages early diagnosis of infertility and informing the patient of her condition. Studies have shown that the later a patient learns of her infertility, the more difficult it is for her to adapt and accept the situation^[11]^.

This case also shows the importance of second opinions in medicine, the underuse of which, despite its importance, was highlighted recently by Halasy *et al*^[12]^.

The medical error that occurred resulted in an incorrect medical intervention, which led to a medicolegal issue against the doctor who did not complete the diagnostic procedures and did not conduct the necessary consultations.

On her last visit, the girl’s school performance was acceptable. Pregnancy can occur through oocyte donation, especially since the girl has a twin sister, but the idea of oocyte donation is not available or illegal in our country, and this has been discussed with the parents.

This case report has been reported in line with the SCARE criteria 2020^[13]^.

## Conclusion

The occurrence of this medical error could be recurring. Therefore, before deciding to perform any surgical procedure to remove a bilateral oophorectomy, the diagnosis of CAIS should be carefully verified by the gold standard, and a clear indication for the removal of the glands should be established after confirming the diagnosis. Therefore, consulting a pediatric endocrinologist and neonatologist is very necessary. This is because surgical errors in such cases are irreparable.

## Data Availability

No new data were generated or analyzed during this study.
